# The role of Toll-like receptor signaling pathways in cerebrovascular disorders: the impact of spreading depolarization

**DOI:** 10.1186/s12974-020-01785-6

**Published:** 2020-04-07

**Authors:** Rezan Ashayeri Ahmadabad, Maryam Khaleghi Ghadiri, Ali Gorji

**Affiliations:** 1 Shefa Neuroscience Research Center, Khatam Alanbia Hospital, Tehran, Iran; 2 Department of Neurosurgery, Westfälische Wilhelms-Universität Münster, Münster, Germany; 3 Epilepsy Research Center, Westfälische Wilhelms-Universität Münster, Münster, Germany; 4 Department of Neurology, Westfälische Wilhelms-Universität Münster, Münster, Germany; 5 Neuroscience research Center, Mashhad University of Medical Sciences, Mashhad, Iran

**Keywords:** Stroke, Spreading depolarization, Toll-like receptors, Subarachnoid hemorrhage, Inflammatory mediators, Chemokines, Brain

## Abstract

Cerebral vascular diseases (CVDs) are a group of disorders that affect the blood supply to the brain and lead to the reduction of oxygen and glucose supply to the neurons and the supporting cells. Spreading depolarization (SD), a propagating wave of neuroglial depolarization, occurs in different CVDs. A growing amount of evidence suggests that the inflammatory responses following hypoxic-ischemic insults and after SD plays a double-edged role in brain tissue injury and clinical outcome; a beneficial effect in the acute phase and a destructive role in the late phase. Toll-like receptors (TLRs) play a crucial role in the activation of inflammatory cascades and subsequent neuroprotective or harmful effects after CVDs and SD. Here, we review current data regarding the pathophysiological role of TLR signaling pathways in different CVDs and discuss the role of SD in the potentiation of the inflammatory cascade in CVDs through the modulation of TLRs.

## Introduction

Cerebrovascular diseases (CVDs) include conditions and disorders that affect blood supply to the brain [1]. CVDs largely include transient ischemic attack (TIA), acute ischemic stroke (AIS), intracerebral hemorrhage (ICH), subarachnoid hemorrhage (SAH), cerebral venous sinus thrombosis (CVST), arteriovenous malformation, and numerous inherited cerebral vasculopathies [2]. Among these, AIS is the second leading cause of death and third leading cause of disability worldwide [3, 4].

Numerous studies indicate that inflammation can be induced by hypoxia. It has been shown that inflammatory markers, like interleukin-6 (IL6) and C-reactive protein, increased in healthy climbers at the altitude of more than 3400 m [5], in organ graft patients [6], and in mice with short-term exposure to low oxygen environments [7]. Complete or partial disruption of regional cerebral blood flow (rCBF) results in an ischemic insult, which through different mechanisms finally lead to cell injury and death [8]. Cell damage due to ischemic injury induce a local inflammation in response to endogenous molecules which are released from cell debris, which in turn exacerbate the ischemic injury [9]. The activation of resident microglia, the recruitment of macrophages and leukocytes, and the production of pro-inflammatory cytokines and other inflammatory mediators are the main part of local inflammatory responses to ischemic injury [10]. Under ischemic conditions, ischemic-induced mediators provoke transcription of various genes, such as nuclear factor κB (NF-κB) and Toll-like receptors (TLRs), which play a crucial role in the modulation of immune system [7, 11]. NF-κB, the main transcription factor of TLRs, activates via the secretion of various cytokines and chemokines [12]. The activation of TLRs pathway triggers various inflammatory processes. TLRs are cell membrane receptors with a fundamental role in the recognition of pathogen molecules, such as bacterial lipopeptides, flagellin, and lipopolysaccharides [10, 13]. The activation of TLRs can also be modulated in response to different types of cytokines, like chemokines, interferons, and interleukins, which are presented in the vicinity of ischemic brain tissue [14].

Accumulating evidence suggest that TLRs play a crucial role in the pathogenesis of AIS [15, 16], ICH [17, 18], and SAH [19]. TLRs activation following ischemic insults could be pathogenic or neuroprotective, depending on the context. TLRs signals, via the recruitment of specific molecules, lead to the activation of inflammatory cytokines that can worsen the ischemic insult [20]. In contrast, activation of TLRs signaling pathway before cell damage can protect the cells from further damage, a process called pre-conditioning [21]. Moreover, several studies have shown the modulatory effects of TLRs agonists/antagonists on the pathophysiologic procedures of different CVDs [21–24]. Modulation of various TLRs subtypes, particularly TLR3 and TLR4, could affect CVD-induced brain injury and their neurological deficits. The role of TLR4/NF-κB signaling pathway in ICH has been reported [25]. It has been suggested that drugs target TLR signaling pathways that offer novel opportunities for exerting neuroprotective effects against various CVDs [14, 21].

Spreading depolarization (SD) is a transient or sustained negative shift of the DC potential, which propagates over the cerebral grey matter at a slow rate of a few mm/min [26–28]. The cerebral blood flow response to SD consists of at least three distinct elements: an initial transient hypoperfusion, a dominant transient hyperemia, and a long-lasting oligemia [29]. The rCBF response to SD modulates by the metabolic condition of the tissue [29, 30]. In patients, terminal SD occurs during cardiac arrest [31, 32] and the development of brain death [33]. SD occurs in patients suffering from various CVDs, such as AIS [34, 35], ICH [36], and SAH [35, 37]. In fact, SD occurs in the ischemic core during the development of focal ischemic insults [38]. In focal cerebral ischemia, SD not only occurs in the ischemic core and penumbra but also they can invade the adequately perfused surrounding tissue. SD wave changes along its spread from the core to the penumbra to the surrounding tissue from a long-lasting harmful to a short-lasting harmless event [39]. The SD continuum is characterized by the continuity of depolarization waves that convert from persistent to progressively shorter ones as they spread from an initial ischemic event. Within a few minutes of an acute severe ischemic event, the onset of persistent depolarization leads to the breakdown of ion homeostasis and induction of cytotoxic edema [39]. The pharmacological profile alters along the SD continuum. In the ischemic periphery, SD can be inhibited by the n-methyl-d-aspartate (NMDA) receptor antagonists [40].On the contrary, blocking of NMDA receptor is progressively less capable of ceasing SD in increasingly more ischemic tissue [41].

The SD continuum can exacerbate the ischemic insults due to the induction of the cytotoxic edema of the brain’s grey matter and lead to further inflammatory responses in CVDs [42]. The association between the occurrence of SD in CVDs and poor clinical outcomes has led to the nomination of SD as a potential biomarker for assessment of brain injury [43]. SD induces a severe vasoconstriction that under specific conditions leads to an instantaneous hypoperfusion (spreading ischemia) and exacerbates cell injury and death [44–46]. It is important to note that SD induces an increase in cerebral blood flow that is variably followed by a mild decrease in normal tissue. The predominant response is thus an increase rather than a decrease in such tissue. By contrast, SD can induce a severe decrease of cerebral blood flow variably followed by an increase of cerebral blood flow in tissue at risk (= inverse hemodynamic response or spreading ischemia) [47, 48]. Thus, the predominant response is a decrease in tissue at risk. Furthermore SD initiates inflammatory responses that contribute to cell damage and death in juvenile rats mainly via the modulation of astrocytes [44, 49–51]. However, according to another study that were done in adult rats, SD can only induce irreversible neuronal injury in the tissues at risk [52]. This data also confounding but reveals that SDs could be harmful to juvenile neuronal tissue under specific slice preparation that is differ from what has been recognized about their effect on adult neuronal tissue [50, 51]. Furthermore, it has been shown that repetitive high mobility group box 1 (HMGB1)–TLR2/4 signaling pathway is a mediator of repetitive SD-induced microglial activation, which may be contributing to neuroinflammatory responses following SD [53]. In this review, after a brief overview of the TLR signaling pathways, we accumulate all evidence regarding the implications of TLRs in CVDs. Then, we discuss the role of SD in the potentiation of the inflammatory cascade in CVDs through the modulation of TLRs.

## General aspects of Toll-like receptor signaling pathways

**Fig. 1:**
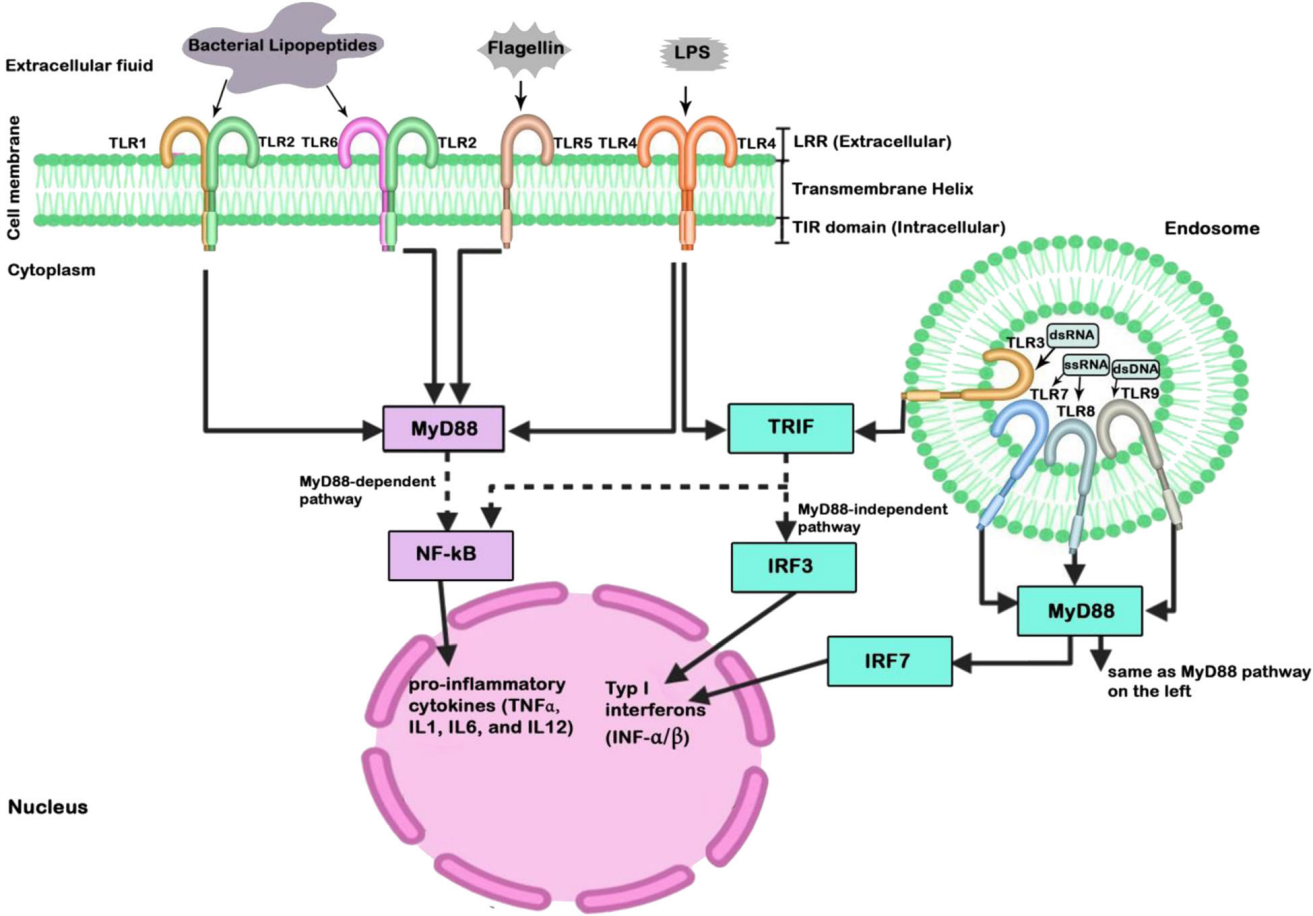
Schematic diagram of Toll-like receptor (TLRs) signaling pathways. TLRs are expressed in neurons, microglia, astrocytes, oligodendrocytes, and neural stem cells. LPS, lipopolysaccharide; LRR, leucine-rich repeat; dsDNA, double-stranded DNA; dsRNA, double-stranded RNA; ssRNA, single-stranded RNA; TIR, toll/IL1 receptor; IRF, interferon regulatory factor; MyD88, myeloid differentiation primary response 88; TRIF, TIR-domain-containing adapter inducing IFN-β; IFN, interferon

TLRs are transmembrane receptors that have critical role not only in innate immune response, but in inflammation and immune cell regulation as well as cell survival and proliferation [54–57]. To date, 13 TLRs have been recognized, including TLR1 through TLR13. However, human cells do not express the last 3 of them [58]. As shown in Fig. 1, TLR1, TLR2, TLR4, TLR5, and TLR6 are localized in the plasma membrane, and TLR3, TLR7, TLR8, and TLR9 are expressed within endosomes [59]. TLRs are expressed in neurons, microglia, astrocytes, oligodendrocytes, and neural stem cells [60]. TLRs detect exogenous molecules, like bacterial lipopeptides, flagellin, lipopolysaccharides, double-stranded DNA (dsDNA), double-stranded RNA (dsRNA), and single-stranded RNA (ssRNA) as well as endogenous molecules called damage-associated molecular patterns (DAMPs) released from injured cells [11]. TLRs are activated through binding of pathogen-associated molecular patterns (PAMPs), which are entirely expressed by microbial pathogens, and DAMPs that are host molecules released from injured cells [61]. This incident will result in a cascade of events through two main TLR-associated signaling pathways that lead to activation of different transcription factors. The first one is myeloid differentiation primary response 88 (MyD88), which in one hand, acts as an adapter protein to activate NF-κB via almost all TLRs, except TLR3, and on the other hand, activates and interacts with transcription factor IRF-7. The latter pathway forms a bridge between the MyD88-dependent and independent pathways. Furthermore, TLR9-MyD88-IRF7 signaling has a critical implication in dendritic cell-based immune responses, as it promotes robust type I interferon induction in these cells [62]. The second pathway is TLR-domain-containing adapter-inducing interferon-β (TRIF) that is associated with TLR3 and TLR4 to activate NF-κB and interferon regulatory factor (IRF) [63]. TLR4 activates both the MyD88-dependent and the MyD88-independent pathways. NF-κB is a transcription factor leading to produce pro-inflammatory cytokines (TNFα, IL1, IL6, and IL12), whereas IRF is an immunomodulatory transcription factor which leads to the production of type I interferons (IFN-α/β) and develops an innate antiviral response [24]. Another important ligand for TLRs are HMGB1, which is a nuclear protein secreted by both immune cells and necrotic cells after hypoxic-ischemic (H-I) damage. HMGB1 mostly binds to TLR2 and TLR4 to induce inflammation through NF-κB signaling pathway [64]. Although HMGB1 has been proposed as a target for cancer therapy, increasing evidence suggests its possible biphasic role in CVDs [65].

### The role of Toll-like receptors in cerebral vascular diseases

TLR signaling pathways implicated in the pathogenesis of several neurological disorders, such as multiple sclerosis [66], epilepsy [67], Alzheimer’s disease [68, 69], Huntington’s disease [70], Parkinson’s disease [71], Lewy body dementia [72], amyotrophic lateral sclerosis [73, 74], migraine headache [75], the central nervous system infections [76], and traumatic brain injury [77]. Modulation of TLRs has been shown to contribute to CVDs, including AIS, ICH, SAH, and CVST (Table 1).

**Table 1:** Brief overview of the association between Toll-like receptors and cerebral vascular diseases

CVDs				
TLRs	AIS	ICH	SAH	CVST
TLR2	Moderator for leukocytes and microglial infiltration and neuronal death [78, 79]	–		Activated in antiphospholipid syndrome [80]
TLR3	Neuroprotective and anti-inflammatory effects on SD-induced neuroinflammation [63] Ischemic tolerance induction by TLR3 ligand poly I:C preconditioning through type I IFN signaling [81]	Neuroprotective and anti-inflammatory effects on SD-induced neuroinflammation [21, 82]		
TLR4	Neuroprotective effect by preconditioning through suppression of cytotoxic TNFα, increasing IRFs and production of type I interferons [78, 79] Induction and evolution of atherosclerosis through NF-κB pathway that produce inflammation [78, 79]	Increasing levels of inflammatory factors, DNA damage, and neuronal degeneration in perihematomal region [82]	Activation by Heme product through the MyD88 and TRIF pathways [83]	Activated in antiphospholipid syndrome [80]
TLR7	Neuroprotective effect by preconditioning with TLR7 ligand, gardiquimod, reduction in infarct size, and a better functional outcome independent of TNFα and dependent on interferon [84]	–		
TLR8	Activation causes worsening of ischemic brain injury [85]	–		–
TLR9	Neuroprotective effect by preconditioning through suppression of cytotoxic TNFα, increasing IRFs, and production of type I interferons [20]	–		Deletion is associated with larger venous thrombosis and increased leukocyte infiltration [80]

*AIS* acute ischemic stroke, *APS* antiphospholipid syndrome, *ICH* intracerebral hemorrhage, *CVD* cerebral vascular disease, *CVST* cerebral venous sinus thrombosis, *SAH* subarachnoid hemorrhage, *SD* spreading depolarization, *TNF* tissue necrosis factor*, IFNA* interferon-α/β receptor, *TLR* Toll-like receptor

### The role of Toll-like receptors in acute ischemic stroke

Atherosclerosis, which is the main cause of AIS, is an inflammatory process with immune response during initiation and progression of the disease [86]. The endothelium is a main contributor of vascular integrity due to its anti-inflammatory property. Evidence shows that endothelial dysfunction is the first measurable step of atherothrombosis formation [87]. In this regard, TLRs and particularly TLR4, which are found in the endothelial cell plasma membrane, have a critical role in the induction and the evolution of atherosclerosis [87–89]. Various cell types in the atherosclerotic vessel wall express TLR4, including neutrophils, macrophages, endothelial cells, fibroblasts, and dendritic cells [90–93]. Activation of TLR4 produce cytokines, which influence multiplication and migration of vascular smooth muscle cells and higher expression levels of MMP-2 and MMP-9 [94]. Monocytes and T lymphocytes will be recruited to the arterial TLR4 ligands during the initial phases of atherogenesis. This requires expression of adhesion molecules on the endothelium, which regulates transcription of TLRs through modulation of NF-κB values [94].

AIS activates the TLR signaling pathway, leads to the production of a plenty of inflammatory mediators, and triggers secondary inflammation damages. However, a mild ischemic insult can lead to TLR ischemic tolerance and decrease brain injury through the inhibition of the TLR4/NF-κB and TLR2 signaling pathway and the activation of IRF3 signaling: a process points to the beneficial effect of MyD88 signaling pathway [95]. In another word, exposure to a minor cerebral ischemia enhances neuronal tolerance to subsequent injury and shifts cellular signaling from NF-κB pathway to IRF3, which produces IFN-b, one of the final products of IRF3 signaling pathway with neuroprotective effects. Administration of a low dose of TLR2, TLR3, TLR4, TLR7, or TLR9 ligand before H-I insult promotes neuroprotection and reduces the infarct volume in animal experimental models [20]. Systemic administration of low doses of lipopolysaccharide (LPS), a TLR4 ligand, a cell wall component of gram–negative bacteria, to hypertensive rats caused tolerance to subsequent brain ischemia induced by middle cerebral artery occlusion [96]. Several other animal models of AIS have also revealed the LPS-induced tolerance to brain ischemia [97–99]. The mechanism by which LPS enhances the tolerance to cerebral ischemia could be attributed to the suppression of cytotoxic TNFα signaling following AIS. Once the TLRs reprogrammed, their response to subsequent brain ischemia could be increasing IRFs and production of type I interferons. Based on a similar mechanism, TLR9 ischemic tolerance following stimulation by cytosine-guanine oligodeoxynucleotides (CpG-OdN) exhibited the neuroprotective effect [100–102]. CpG-OdN inhibits cerebral ischemic injury and reduces the lesion volume via a PI3K/Akt-dependent pathway [103]. Moreover, the role of TNFα signaling in the preconditioning with TLR ligands has been demonstrated. Administration of TNFα itself reprogrammed the cell structure in favor of the remodeling of the inflammatory response to the subsequent ischemia [100–102]. Interestingly, CpG-OdN-induced preconditioning in a mouse model of AIS changed the genomic response to stroke in the circulating leukocytes and the brain cells [102]. In addition, it has been shown that TLR2 ischemic tolerance may attenuate the brain lesion after AIS. Inhibition of TLR2 signaling pathway regulates leukocytes and microglial infiltration and the subsequent neuronal death after mild AIS [78, 87, 104, 105]. Inhibition of TLR4 could attenuate the inflammation and H-I damages through blockade of tissue-type plasminogen activator-induced hemorrhagic transformation [106] as well as enhancement of the ratio of alternative neutrophils [15]. It has been shown that TLR4-deficient mice have significantly less tolerance to H-I insults than wild-type mice, possibly via the lesser expression of TNFα, cyclooxygenase-2 (COX-2), and NF-κB [107]. An experimental study has shown that the western diet provokes TLR4-induced endothelial dysfunction and suggesting a potential role of TLR4-related inflammation in increasing the risk of AIS [108].

One of the most important ligand for TLRs, especially TLR2 and TLR4, is HMGB1. Both experimental and clinical studies indicate that HMGB1 is released from injured brain tissues as well as activated microglia within the ischemic tissues and activates an early inflammatory response after AIS [109]. HMGB1signals via TLR2 and TLR4 signaling activate the NF-κB pathway and induce a proinflammatory response [110]. Several studies have shown that plasma levels of HMGB1 increase in ischemic stroke and correlate with poor outcome [111–113]. Despite the potential inflammatory role of HMGB1 in the acute phase of stroke, there is some evidence in favor of its immunomodulatory action in the delayed phase [65]. HMGB1 has been suggested as a potential biomarker for prediction of the AIS prognosis [110].

In addition to TLR4 and TLR2, evidence suggests the implication of TLR7, TLR8, and TLR9 in the neuroinflammtory responses induced by H-I insults. Application of the TLR7 agonist Gardiquimod before middle cerebral artery occlusion in mice reduces infarct volume as well as functional deficits: a neuroprotective effect independent of TNF and dependent on interferon [84]. A greater expression of TLR7 and TLR8 was associated with a higher inflammatory response and a worse outcome in patients with AIS. The expressions of TLR7 and TLR8 were correlated with IL6 and IL1β values during the first week of AIS. In addition, the expression of TLR8 was correlated with cerebral infarct volumes [85]. Furthermore, it has been shown that exogenous preconditioning with TLR2, TLR4, and TLR9 ligands prior to the occurrence of AIS provides neuroprotection [114].

### The role of Toll-like receptors in intracerebral hemorrhage

Recent investigations have shown that inflammation to some degree is responsible for secondary brain damage after ICH [25]. It has been suggested that the activation of TLRs, particularly TLR4, contributes to the microglial phagocytosis and consequently to the development of inflammatory brain injury in ICH [115]. Induction of ICH in TLR4-deficient transgenic mice has been revealed a significant reduction of perihematomal inflammation and decreased recruitment of inflammatory cells associated with the promotion of functional recovery, pointing to the role of TLR4 in poor functional outcomes in ICH [85]. In addition to TLR4, higher expression of TLR2 in both monocytes and neutrophils is associated with a larger lesion volume and poor outcomes in patients with ICH [116]. Repetitive intraperitoneal administration of TAK-242, a TLR4 antagonist, in an ICH mouse model remarkably reduced brain water capacity, peripheral inflammatory cell infiltration, the values of inflammatory mediators, and neurological deficit scores. The expressions of TLR4 downstream signaling molecules, including MyD88, TRIF-inducing interferon-beta IκBα, and NF-κB, were significantly downregulated in these mice [25].

Iron plays a harmful role in the pathogenesis of ICH and causes oxidative damage, impaired cognitive function, and poor prognosis [117]. Heme, a component of hemoglobin, acts on TLRs and leads to downward activation of NF-κB signaling, promotes inflammation, and aggravate ICH-induced neurological deficit [25, 82]. Heme-induced brain inflammatory damages could be initiated by the activation of microglia and subsequent release of proinflammatory mediators [118]. Hemoglobin is a strong activator of microglia through activation of TLRs [18]. Hemoglobin induces inflammatory responses following ICH via assembly of TLR2/TLR4 heterodimers, which is a MyD88-dependent process [119]. Heme oxygenase knockout mice have shown a significant decrease in microglia activation, free radical levels, and leukocyte infiltration after ICH [120]. TLR4- and MyD88-deficient mice exhibited an improved brain iron efflux after ICH, and application of a TLR4 antagonist significantly reduced brain iron values associated with promotion of cognitive functions [121]. Sparstolonin B, a *Sparganium stoloniferum* derivative that selectively inhibits TLR2/TLR4, could reduce secondary inflammatory injury after ICH. Sparstolonin B dose dependently inhibited NF-κB activity through TLR2/TLR4 heterodimer formation and attenuated inflammation after ICH [122]. Furthermore, it has been shown that inflammatory injury of the choroid plexus epithelium due to activation of TLR4-NF-κB signaling pathways contributes to increased secretion of cerebrospinal fluid following intraventricular hemorrhage [123].

The activation of TLRs can also interact with the downstream spleen tyrosine kinase (Syk) and trigger the release of several inflammatory cytokines, such as IL-1β [124]. This pathway plays a role in traumatic brain injury [125], AIS [126, 127], and ICH [128]. Inhibition of the macrophage-inducible C-type lectin/Syk pathway decreases the release of proinflammatory cytokines and attenuates neurological deficits after ICH [128].

### The role of Toll-like receptors in subarrachnoid hemorrhage

Rupture of saccular aneurysms accounts for the majority of SAH, although the reason for non-aneurysmal SAH is divergent [129]. Hypothesis for the role of inflammation in SAH arises from several investigations, which focused on vasospasm as the main mechanism for the delayed neurologic deficit. As the delayed ischemic neurologic deficit after SAH can occur independently of angiographic vasospasm, it was suggested that the other mechanisms, such as neuroinflammation, may contribute to this distinctive syndrome of cerebral ischemia [130]. Experimental studies have revealed an upregulation of TLR4-NF-κB signaling pathway in early brain injury following SAH [131]. Toll-like receptor signaling and NF-κB transcription factor binding sites were significantly enhanced after SAH [132]. Furthermore, experimental studies revealed a correlation between the upregulation of TLR4 with the development of cerebral vasospasm after SAH [133]. Enhancement of TLR4 expression on the endothelial cell layer in the wall of the human cerebral aneurysm has been suggested that TLR4 may play an important role in the formation of brain aneurysm [134, 135]. Tenascin-C, a matricellular protein, initiates a severe cerebral arterial constriction following SAH [19]. Application of Tenascin-C enhances the expression of TLR4 in the smooth muscle cell layer of the affected cerebral artery and increases the SAH-induced subarachnoid as well as systemic inflammatory responses [19]. Administration of TLR4 antagonist LPS-RS inhibits Tenascin-C-induced cerebral vasospasm and upregulation of TLR4 in rats [136]. Dendritic cells, antigen-presenting cells that regulate the adaptive immune response, express several TLRs. The frequency of myeloid dendritic cells producing TNF-α after application of TLR-3 agonist poly I:C significantly decreased in the SAH patients. In addition, myeloid dendritic cells producing administration of TLR-3 and 4 agonists decreased in the SAH patients compared to healthy subjects [137].

Common mechanisms may underlie the induction of inflammatory processes after ICH and SAH. The breakdown of hemoglobin and the release of heme after SAH could lead to brain inflammatory injury. Heme activates TLR4-induced neuroinflammatory process through the MyD88 and TRIF pathways [118]. Microglia and the TLR4 pathway may play an important role in vasospasm and neuronal apoptosis after SAH. Using TLR4, TRIF, and MyD88 knockout mice, it has been shown that the activation of TLR4-MyD88 pathway is essential for neuronal apoptosis and vasospasm during the early phase of SAH, whereas neuronal damage and vasospasm in the late phase of SAH were TLR4-TRIF dependent. Microglial cells play a crucial role in SAH-induced vasospasm in both the early and late phases of SAH [83]. The effects of two selective TLR4 inhibitors on SAH-induced disruption of the blood rain barrier (BBB) were assessed in a SAH experimental model [138, 139]. Intracerebroventricular application of these selective TLR4 antagonists, IAXO-102, and TAK-242, in male mice with SAH significantly reduced post-SAH neurological deficits, the BBB disruption, and brain edema, leading to a better survival rate [139].

Furthermore, it has been suggested that peroxisome proliferator-activated receptor gamma (PPAR-γ) receptors play a modulatory role in SAH-induced inflammatory responses through the TLR4 pathway [140]. Activation of PPAR-γ receptors is shown to efficiently inhibit TLR4 pathway [141]. Rosiglitazone, a PPAR-γ agonist, inhibited the SAH-induced inflammatory injury as well as cerebral vasospasm in basilar arteries via suppression of the TLR4 signaling pathway in an animal experimental SAH model [142]. Pannexin-1 channels, a member of gap junction proteins involved in the innate and acquired immune system, could also contribute to the induction of inflammation and cognitive dysfunction of SAH via the TLR2/TLR4/NF-κB-mediated signaling pathways. Pannexin-1 channels protein gene knockdown significantly reduced the expression values of TLR2/4/NF-κB signaling pathway in the neocortex and improved cognitive and memory deficits in a rat SAH model [143].

### The role of Toll-like receptors in cerebral venous sinus thrombosis

The role of TLR9 signaling pathway in venous thrombus resolution was investigated in a mouse model of CVST [144]. It has been found that mice with the deletion of TLR9 had significantly a larger venous thrombosis and increased leukocyte infiltration compared with wild-type mice. Although MyD88 is the intracellular signaling pathway of TLR9, mice lacking MyD88 had no greater venous thrombus resolution than wild-type mice. Application of TLR9 agonist was associated with smaller venous thrombosis in this model. It has been shown that the TLR pathway mainly through TLR2 and TLR4 is activated in patients with antiphospholipid syndrome, suggesting a biomarker role of TLRs in this syndrome [80]. Moreover, a significant rise in the gene expression of TLR2 and TLR4 was observed in peripheral mononuclear cells that mediate antiphospholipid antibodies induced vascular abnormalities. Hence, targeting these receptors as a therapeutic option to prevent the thrombotic effects of antiphospholipid antibodies in antiphospholipid syndrome could be the subject of interest in the future studies.

### Contribution of spreading depolarization to inflammatory response following cerebral vascular diseases

SD was discovered over 75 years ago by Aristides Leão in the course of his investigations on the initiation and propagation of epileptiform burst discharges [145]. Over the past decades, compelling evidence has accumulated to demonstrate the occurrence of SD in the brain of patients suffering from CVDs [29, 146, 147]. Using intracranial recordings, the occurrence of repetitive SD has been shown in patients with AIS [34, 35], ICH [148, 149], and SAH [35, 37, 48]. There are numerous experimental and clinical data suggesting that SD aggravate tissue injury and neurological outcomes of CVDs [150, 151]. The normal response in healthy tissue is a brief increase of rCBF (spreading hyperemia) followed by a prolonged decrease (spreading oligemia). The altered or pathological response in tissue at risk is a steep and prolonged fall in rCBF variably followed by a prolonged hyperemia [47, 48, 152–154]. Furthermore, the propagation of SD is associated with an increase of glucose utilization and O_2_ consumption as well as with marked increases in local energy requirements to restore ion homeostasis [155, 156]. The combinations of reduced rCBF enhanced energy demand, mitochondrial depolarization, and a massive Ca^2+^ influx as well as O_2_, glucose, and ATP deprivation following SD augments ischemic conditions and exacerbates cell damage and death in CVDs [28, 157, 158]. SD clusters with prolonged depression phases promote delayed ischemic brain damage after SAH and malignant ischemic stroke [35, 159], possibly via reduction of O_2_ supply and enhancement of O_2_ consumption [160]. In experimental models of brain ischemia, the occurrence of SD in the ischemic penumbra and outside of the penumbra zone leads to cell damage and death as well as enlargement of the ischemic core and the infarct zone [161–163]. Clinical investigations reveal a strong association between repetition rate of spontaneous SD and delayed ischemic neurological deficits [35]. In addition to the mismatch between supply and demand [164], lowering the seizure threshold [148, 165], disruption of the BBB [166], and SD-induced inflammatory responses contributes to tissue damage and clinical outcomes of patients with CVDs [167]. SD can affect both ischemic preconditioning and ischemic-induced brain injury after CVDs [107, 114].

These diverse effects on ischemic preconditioning and ischemic-induced brain injury could be dependent on the vicinity of the damaged cerebral area. SD is the mechanism of the cytotoxic edema [31]. The SD-induced cytotoxic edema occurs very early after the onset of ischemia, which is clearly not beneficial to the brain. However, in healthy tissue surrounding the ischemic zone, SD could have beneficial effects via upregulation of growth factors, stress response proteins, and potentially beneficial inflammatory mediators [168, 169]. This may enhance synaptic plasticity [170], boost regeneration process [171, 172], and reduce the vascular steal effect on ischemic zones through the physiological oligemia [173]. Furthermore, it seems that TLR signaling pathway may exert controversial effects in early stages of various CVDs.TLR-4 in AIS exerts neuroprotective effect via ischemic preconditioning, while it is detrimental in ICH. The beneficial effect of SD on these pathological conditions could be dependent on the extent of ischemic injury in the implicated neuronal tissues [172].

Several investigations indicate that both SD and CVDs are potent modulators of immediate early genes (such as c-JUN, JUN B, JUN D, c-Fos, Fos B, and KROX-24) [174–176]. Within the same time course of immediate early gene, SD and CVDs induce heat shock proteins [177, 178]. Furthermore, upregulation of neurotrophic mediators is associated within early phases of SD and CVDs [169]. These early alterations may contribute to the preconditioning phenomenon in CVDs. The activation of immediate early genes and heat shock proteins as well as upregulation of neurotrophic cascade may exert neuroprotective effects and prevent post-ischemic inflammation and neuronal injury [179, 180]. However, delayed SD-induced inflammatory cascades seem to be harmful for neuronal tissues.

Using animal models for SD and brain ischemia, it has been shown that an increased prostaglandin and subsequently COX-2 production was observed within hours after SD and brain ischemia in perifocal neocortex, the hippocampus, and the striatum as well as in endothelial cells in the infarct core, which may amplify the ischemic inflammation and cell death via enhancement of edema and delivery of pro-inflammatory cells into the ischemic brain [181]. However, the role of COX-2 in SD-induced inflammatory responses could be a more complex issue. Although COX-2 is upregulated by SD, the selective inhibition of COX-2 has proven to be ineffective at altering the DC signature or the rCBF response to SD, at least within the first hours after SD induction or ischemia onset [182]. SD-induced hyperexcitability activates microglia and astrocytes and promotes the release of pro-inflammatory mediators, such as TNFα, reactive oxygen species (ROS), and interferon [183–185], which may lead to neuronal tissue damage. Oxidative stress that leads in the enhancement of ROS can damage DNA and potentiate neuronal injury and death following H-I injury [186]. SD in intact rodent brain results in microglia activation and a strong induction of the pro-inflammatory cytokines TNF-α and IL-1β [187]. Furthermore, the expressions of IL-1α, IL-2, IL-4, IL-6, IL-10, IFN-γ, and TNF-α proteins markedly enhanced after SD with localization of IL-1α and IFN-γ to microglia in hippocampal organotypic cultures [168]. Increased potassium concentration during SD augments the production of glial inflammatory factors nitric oxide and TNF-α, which exert toxic effects on neuronal tissues [188]. Furthermore, SD-induced inflammatory response is associated with the activation of macrophages and mast cells and a rise in cytokine levels [189, 190].

Numerous amounts of evidence suggest that SD-induced inflammatory responses contribute to cell damage and death in both in hypoxic and intact conditions. SD upregulates a set of inflammation-related genes [183], many of those, like x-box binding protein-1 and interferon regulatory factor-1, contribute to cell injury and apoptosis in patients with CVDs [180, 191, 192]. SD-related caspase activation, as a part of inflammatory response to neuronal stresses [193], is associated with a widespread apoptosis and cell damage in the neocortex and hippocampus [44]. SD-induced cell damage and apoptosis are correlated with the activation of synaptic plasticity and higher expression levels of glutamatergic NMDA and AMPA receptors [46]. Activation of caspase cascade could be due to SD-induced release of different cytokines, such as IFN-γ and TNF-α [168, 194].

Accumulating evidence points to the role of TLRs in SD-induced cell damage and apoptosis in neuronal tissues. Experimental studies revealed that SD induces a marked release of HMGB1, an important ligand of TLR2 and TLR4 [195]. Repetitive SD leads to the activation of microglia associated with an increased transcription activity of the HMGB1 receptor genes, TLR2 and TLR4. Furthermore, an enhanced expression of the lysosomal acid hydrolase cathepsin D accompanied by microglial hypertrophy has been reported after repetitive SD, indicating a higher microglia lysosomal phagocytic activity following SD [53]. In addition to microglia, astrocytes may play a role in TLR-related inflammatory conditions after SD. Induction of reactive astrocytosis has been reported in different CVDs [196] as well as after SD [197], which may potentiate neuroinflammation through the production of inflammatory mediators, such as ROS, chemokines, and cytokines [198]. SD significantly increases the expression of TLR4 and TLR3 in addition to TNF-α, IL-6, and IL-1β in rat somatosensory cortices. In addition, the expressions of TLR4 and TLR3 as well as various pro-inflammatory cytokines markedly increase in cultured astrocytes obtained from SD-treated rat brain [49]. SD leads to parenchymal inflammatory response via neuronal Pannexin1 megachannel opening as well as caspase-1 activation followed by HMGB1 release from neurons and nuclear factor κB activation in astrocytes [193]. Administration of TLR ligands at low concentration prior to H-I insults induces neuroprotection, pointing to the crucial role of these receptors in preconditioning [20]. Application of Poly I:C, a synthetic analogue of dsRNA and TLR3 agonist, reduces cerebral injury through TLR3-mediated prevention of H-I-induced activation of Fas/FADD-mediated apoptotic signaling and microglia caspase-3 and -8 activities [199] as well as the downregulation of TLR4 signaling via TLR3 [200] in animal models of cerebral ischemia. Interestingly, prophylactic application of Poly I:C significantly prevents the production of SD-induced cell injury and attenuates the induction of TNF-α, IFN-γ, and Hsp70 in the brain as well as TNF-α and IL-4 in the spleen. Furthermore, Poly I:C modulates GABAergic neurotransmission, which may inhibit SD-induced neuronal hyperexcitability [201].

## Conclusion

Enormous amounts of experimental and clinical data indicate that SD can modulate neural tissue injury and clinical outcomes in different CVDs. SD contributes to both ischemic preconditioning and exacerbation of cerebral injury and outcome after CVDs. A part of the SD effect on pathological process of CVDs could be due to SD-induced inflammatory responses. TLRs are common signaling pathways in SD- and CVD-induced neuroinflammation. Considering the double-edged role of TLR-related inflammatory responses in both CVDs and SD—beneficial in the acute phase and destructive in the late phase—targeting these receptors may provide novel therapeutic approaches for CVDs and other SD-related disorders. Furthermore, manipulation of TLRs may exert a modulatory effect on SD occurrence and regulate the following pathological conditions.

## Data Availability

Authors confirm that all relevant data are included in the article.

## Abbreviations

AIS: Acute ischemic stroke
BBB: Blood-brain barrier
COX-2: Cyclooxygenase-2
CpG-OdN: Cytosine-guanine oligodeoxynucleotides
CVDs: Cerebrovascular diseases
CVST: Cerebral venous sinus thrombosis
DAMPs: Disease-associated molecular patterns
DC: Dendritic cell
dsDNA: Double-stranded DNA
dsRNA: Double-stranded RNA
H-I: Hypoxic-ischemic
HMGB1: High mobility group box 1
ICH: Intracerebral hemorrhage
IFN: Interferon
IL-6: Interleukin-6
IRF: Interferon regulatory factor
LPS: Lipopolysaccharide
MyD88: Myeloid differentiation primary response 88
NFκB: Nuclear factor-κB
PAMPs: Pathogen-associated molecular patterns
PPAR-γ: Peroxisome proliferator-activated receptor gamma
rCBF: Regional cerebral blood flow
SAH: Subarachnoid hemorrhage
SD: Spreading depolarization
ssRNA: Single-stranded RNA
TAK-242: Ethyl(6R)-6-[N-(2-chloro-4-fluorophenyl) sulfamoyl] cyclohex-1-ene-1-carboxylate
TIR: Toll/IL1 receptor
TLRs: Toll-like receptors
TRIF: TIR-domain-containing adapter-inducing interferon-β
